# Users Polarization on Facebook and Youtube

**DOI:** 10.1371/journal.pone.0159641

**Published:** 2016-08-23

**Authors:** Alessandro Bessi, Fabiana Zollo, Michela Del Vicario, Michelangelo Puliga, Antonio Scala, Guido Caldarelli, Brian Uzzi, Walter Quattrociocchi

**Affiliations:** 1 IUSS, Pavia, Italy; 2 CSSLab, IMT Lucca, Italy; 3 ISC, CNR, Rome, Italy; 4 NICO, Northwestern University, Evanston, IL, United States of America; University of Warwick, UNITED KINGDOM

## Abstract

Users online tend to select information that support and adhere their beliefs, and to form polarized groups sharing the same view—e.g. echo chambers. Algorithms for content promotion may favour this phenomenon, by accounting for users preferences and thus limiting the exposure to unsolicited contents. To shade light on this question, we perform a comparative study on how same contents (videos) are consumed on different online social media—i.e. Facebook and YouTube—over a sample of 12*M* of users. Our findings show that content drives the emergence of echo chambers on both platforms. Moreover, we show that the users’ commenting patterns are accurate predictors for the formation of echo-chambers.

## Introduction

The diffusion of social media caused a shift of paradigm in the creation and consumption of information. We passed from a mediated (e.g., by journalists) to a more disintermediated selection process. Such a disintermediation elicits the tendencies of the users to a) select information adhering to their system of beliefs—i.e., confirmation bias—and b) to form groups of like minded people where they polarize their view—i.e. echo chambers [1–6]. Polarized communities emerge around diverse and heteorgeneous narratives often reflecting extreme disagreement with respect to the main stream news and recommended practices. The emergence of polarization in online environments might reduce viewpoint heterogeneity, which has long been viewed as an important component of democratic societies [7, 8].

Confirmation bias has been shown to play a pivotal role in the diffusion of rumors online [9]. However, on online social media, different algorithms foster personalized contents according to user tastes—i.e. they show users viewpoints that they already agree with. The role of these algorithms in influencing the emergence of echo chambers is still a matter of debate. Indeed, little is known about the factors affecting the algorithms’ outcomes. Facebook promotes posts according to the *News Feed* algorithm, that helps users to see more stories from friends they interact with the most, and the number of comments and likes a post receives and what kind of story it is—e.g. photo, video, status update—can also make a post more likely to appear [10]. Conversely, YouTube promotes videos through *Watch Time*, which prioritizes videos that lead to a longer overall viewing session over those that receive more clicks [11]. Not much is known about the role of cognitive factors in driving users to aggregate in echo chambers supporting their preferred narrative. Recent studies suggest confirmation bias as one of the driving forces of content selection, which eventually leads to the emergence of polarized communities where users acquire confirmatory information and ignore dissenting content [12–17].

To shade light on the role of algorithms for content promotion in the emergence of echo chambers, we analyze the users behavior exposed to the same contents on different platforms—i.e. Youtube and Facebook. We focus on Facebook posts linking Youtube videos reported on Science and Conspiracy pages. We then compare the users interaction with these videos on both platforms.

We limit our analysis to Science and Conspiracy for two main reasons: a) scientific news and conspiracy-like news are two very distinct and conflicting narratives; b) scientific pages share the main mission to diffuse scientific knowledge and rational thinking, while the alternative ones resort to unsubstantiated rumors.

Indeed, conspiracy-like pages disseminate myth narratives and controversial information, usually lacking supporting evidence and most often contradictory of the official news. Moreover, the spreading of misinformation on online social media has become a widespread phenomenon to an extent that the World Economic Forum listed massive digital misinformation as one of the main threats for the modern society [16, 18].

In spite of different debunking strategies, unsubstantiated rumors—e.g. those supporting anti-vaccines claims, climate change denials, and alternative medicine myths—keep proliferating in polarized communities emerging on online environments [9, 14], leading to a climate of disengagement from mainstream society and recommended practices. A recent study [19] pointed out the inefficacy of debunking and the concrete risk of a backfire effect [20, 21] from the usual and most committed consumers of conspiracy-like narratives.

We believe that additional insights about cognitive factors and behavioral patterns driving the emergence of polarized environments are crucial to understand and develop strategies to mitigate the spreading of online misinformation.

In this paper, using a quantitative analysis on a massive dataset (12*M* of users), we compare consumption patterns of videos supporting scientific and conspiracy-like news on Facebook and Youtube. We extend our analysis by investigating the polarization dynamics—i.e. how users become polarized comment after comment. On both platforms, we observe that some users interact only with a specific kind of content since the beginning, whereas others start their commenting activity by switching between contents supporting different narratives. The vast majority of the latter—after the initial switching phase—starts consuming mainly one type of information, becoming polarized towards one of the two conflicting narratives. Finally, by means of a multinomial logistic model, we are able to predict with a good precision the probability of whether a user will become polarized towards a given narrative or she will continue to switch between information supporting competing narratives. The observed evolution of polarization is similar between Facebook and YouTube to an extent that the statistical learning model trained on Facebook is able to predict with a good precision the polarization of YouTube users, and vice versa. Our findings show that contents more than the algorithms lead to the aggregation of users in different echo chambers.

## Results and Discussion

We start our analysis by focusing on the statistical signatures of content consumption on Facebook and Youtube videos. The focus is on all videos posted by conspiracy-like and scientific pages on Facebook. We compare the consumption patterns of the same video on both Facebook and Youtube. On Facebook a *like* stands for a positive feedback to the post; a *share* expresses the will to increase the visibility of a given information; and a *comment* is the way in which online collective debates take form around the topic promoted by posts. Similarly, on YouTube a *like* stands for a positive feedback to the video; and a *comment* is the way in which online collective debates grow around the topic promoted by videos.

### Contents Consumption across Facebook and YouTube

**Fig 1:**
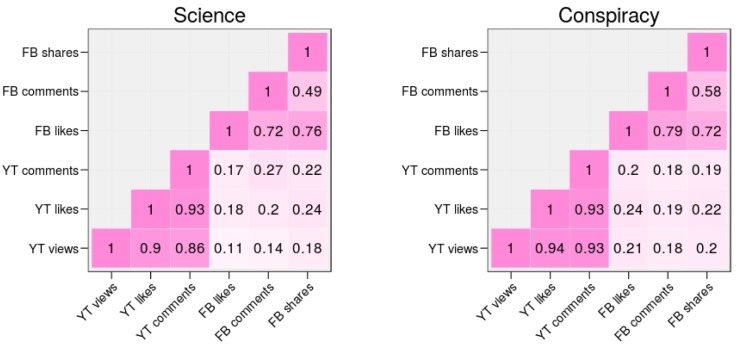
Correlation Matrix. Spearman’s rank correlation coefficients between users’ actions on Facebook posts and the related YouTube videos.

As a preliminary analysis we measure the similarity of the users reaction to the same videos on both platforms. Focusing on the consumptions patterns of YouTube videos posted on Facebook pages, we compute the Spearman’s rank correlation coefficients between users’ actions on Facebook posts and the related YouTube videos (see Fig 1). We find strong correlations on how users like, comment and share videos on Facebook and Youtube. Despite the different algorithm for content promotion, information reverberate in a similar way.

By means of the Mantel test [22] we find a statistically significant (simulated p-value <0.01, based on 10^4^ Monte Carlo replicates), high, and positive (*r* = 0.987) correlation between the correlation matrices of Science and Conspiracy. In particular, we find positive and high correlations between users’ actions on YouTube videos for both Science and Conspiracy, indicating a similar strong monotone increasing relationship between views, likes, and comments. Furthermore, we observe positive and mild correlations between users’ actions on Facebook posts linking YouTube videos for both Science and Conspiracy, suggesting a monotone increasing relationship between likes, comments, and shares. Conversely, we find positive yet low correlations between users’ actions across YouTube videos and the Facebook posts linking the videos for both Science and Conspiracy, implying that the success—in terms of received attention—of videos posted on YouTube does not ensure a comparable success on Facebook, and vice versa. This evidence suggests that the social response to information is similar on different contents and platforms.

**Fig 2:**
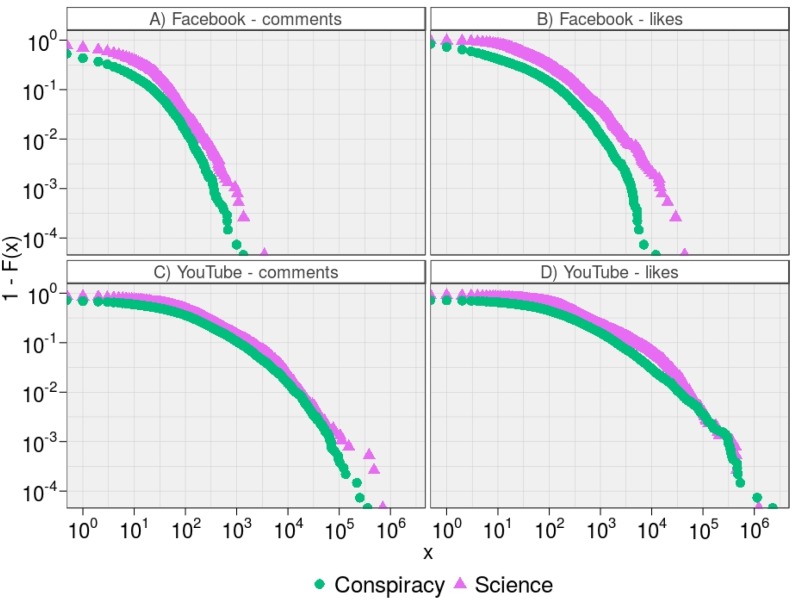
Consumption Patterns of Videos on Facebook and YouTube. The empirical CCDFs, 1 − *F*(*x*), show the consumption patterns of videos supporting conflicting narratives—i.e. Science and Conspiracy—in terms of comments (A and C) and likes (B and D) on Facebook and YouTube.

As a further analysis we focus on the volume of actions to each post. In Fig 2 we show the empirical Cumulative Complementary Distribution Functions (CCDFs) of the consumption patterns of videos supporting conflicting narratives—i.e. Science and Conspiracy—in terms of comments and likes on Facebook and YouTube. The double-log scale plots highlight the power law behavior of each distribution. Top right panel shows the CCDFs of the number of likes received by Science (*x*_*min*_ = 197 and *θ* = 1.96) and Conspiracy (*x*_*min*_ = 81 and *θ* = 1.91) on Facebook. Top left panel shows the CCDFs of the number of comments received by Science (*x*_*min*_ = 35 and *θ* = 2.37) and Conspiracy (*x*_*min*_ = 22 and *θ* = 2.23) on Facebook. Bottom right panel shows the CCDFs of the number of likes received by Science (*x*_*min*_ = 1,609 and *θ* = 1.65) and Conspiracy (*x*_*min*_ = 1,175 and *θ* = 1.75) on YouTube. Bottom left panel shows the CCDFs of the number of comments received by Science (*x*_*min*_ = 666 and *θ* = 1.70) and Conspiracy (*x*_*min*_ = 629 and *θ* = 1.77) on YouTube.

Social response on different contents do not present a significant difference on Facebook and Youtube. Users’ response to content is similar on both platform and on both types of content. Science and Conspiracy videos receive the same amount of attention and reverberate in a similar way.

### Polarized and Homogeneous Communities

**Fig 3:**
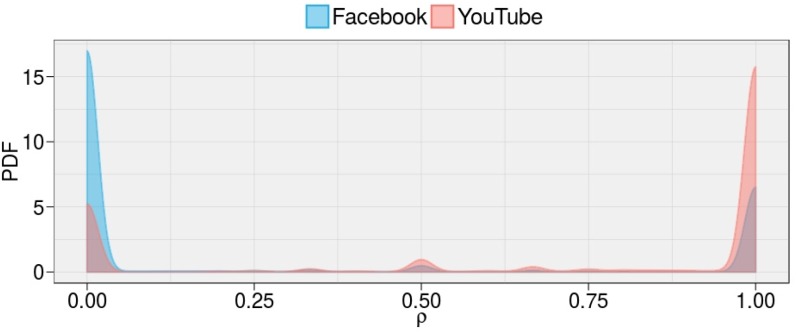
Polarization on Facebook and YouTube. The PDFs of the polarization *ρ* show that the vast majority of users is polarized towards one of the two conflicting narratives—i.e. Science and Conspiracy—on both Facebook and YouTube.

As a secondary analysis we want to check whether the content has a polarizing effect on user. Hence, we focus on the users’ activity across the different type of contents. Fig 3 shows the Probability Density Functions (PDFs) of about 12*M* users’ and on how they distribute their comments on Science and Conspiracy posts (polarization) on both Facebook and YouTube. We observe sharply peaked bimodal distributions. Users concentrate their activity on one of the two narratives. To quantify the degree of polarization we use the Bimodality Coefficient (BC), and we find that the BC is very high for both Facebook and YouTube. In particular, *BC*_*FB*_ = 0.964 and *BC*_*YT*_ = 0.928. Moreover, we observe that the percentage of polarized users (users with *ρ* < 0.05 and *ρ* > 0.95) is 93.6% on Facebook and 87.8% on YouTube; therefore, two well separated communities support competing narratives in both online social networks.

Content has a polarizing effect, indeed, users focus on specific types of content and aggregate in separated groups—echo chambers—independently of the platform and content promotion algorithm.

**Fig 4:**
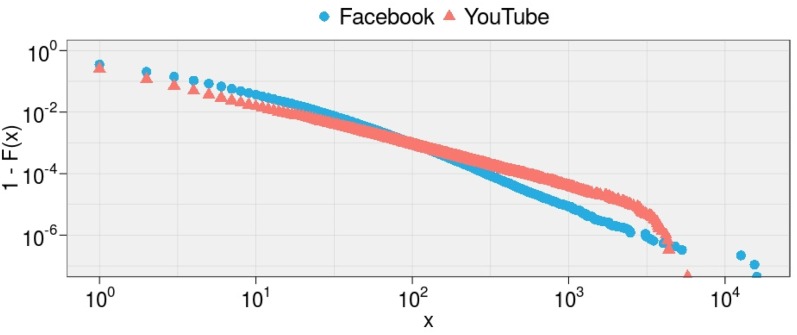
Commenting Activity of Polarized Users. The empirical CCDFs, 1 − *F*(*x*), of the number of comments left by polarized users on Facebook and YouTube.

**Fig 5:**
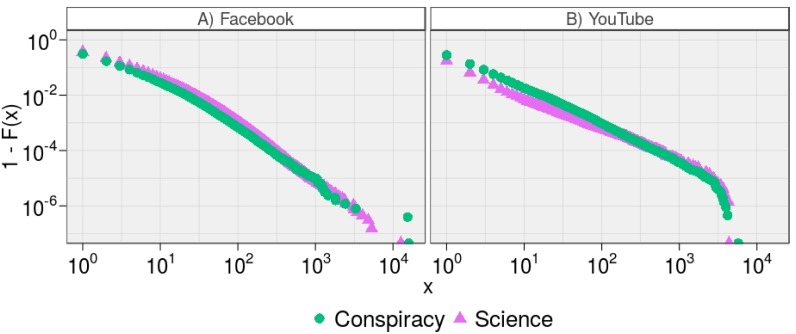
Commenting Activity of Users Polarized towards Conflicting Narratives. The empirical CCDFs, 1 − *F*(*x*), of the number of comments left by users polarized on scientific narratives and conspiracy theories on Facebook (A) and YouTube (B).

To further detail such a segregation, we analyze how polarized users—i.e., users having more than the 95% of their interactions with one narrative—behave with respect to their preferred content. Fig 4 shows the empirical CCDFs of the number of comments left by all polarized users on Facebook and YouTube ($x_{min}^{FB}=8$, *θ*^*FB*^ = 2.13 and $x_{min}^{YT}=17$, *θ*^*YT*^ = 2.29). We observe a very narrow difference (HDI90 = [−0.18,−0.13]) between the tail behavior of the two distributions. Moreover, Fig 5 shows the empirical CCDFs of the number of comments left by users polarized on either Science or Conspiracy on both Facebook ($x_{min}^{Sci}=5$, *θ*^*Sci*^ = 2.29 and $x_{min}^{Con}=4$, *θ*^*Con*^ = 2.31, with HDI90 = [−0.018,−0.009]) and YouTube ($x_{min}^{Sci}=2$, *θ*^*Sci*^ = 2.86 and $x_{min}^{Con}=3$, *θ*^*Con*^ = 2.41, with HDI90 = [0.44, 0.46]). Users supporting conflicting narratives behave similarly on Facebook, whereas on YouTube the power law distributions slightly differ in the scaling parameters.

The aggregation of users around conflicting narratives lead to the emergence of echo chambers. Once inside such homogeneous and polarized communities, users supporting both narratives behave in a similar way, irrespective of the platform and content promotion algorithm.

### Prediction of Users Polarization

Now we want to characterize how the content attract users,—i.e. how users’ polarization evolves comment after comment. We consider random samples of 400 users who left at least 100 comments, and we compute the mobility of a user across different contents along time. On both Facebook and YouTube, we observe that some users interact with a specific kind of content, whereas others start their commenting activity by switching between contents supporting different narratives. The latter—after an initial switching phase—starts focusing only on one type of information, becoming polarized towards one of the two conflicting narratives. We exploit such a regularity to derive a data-driven model to forecast users’ polarizations. Indeed, by means of a multinomial logistic model, we are able to predict the probability of whether a user will become polarized towards a given narrative or she will continue to switch between information supporting competing narratives. In particular, we consider the users’ polarization after *n* comments, *ρ*_*n*_ with *n* = 1, …, 100, as a predictor to classify users in three different classes: Polarized in Science (*N* = 400), Not Polarized (*N* = 400), Polarized in Conspiracy (*N* = 400).

**Fig 6:**
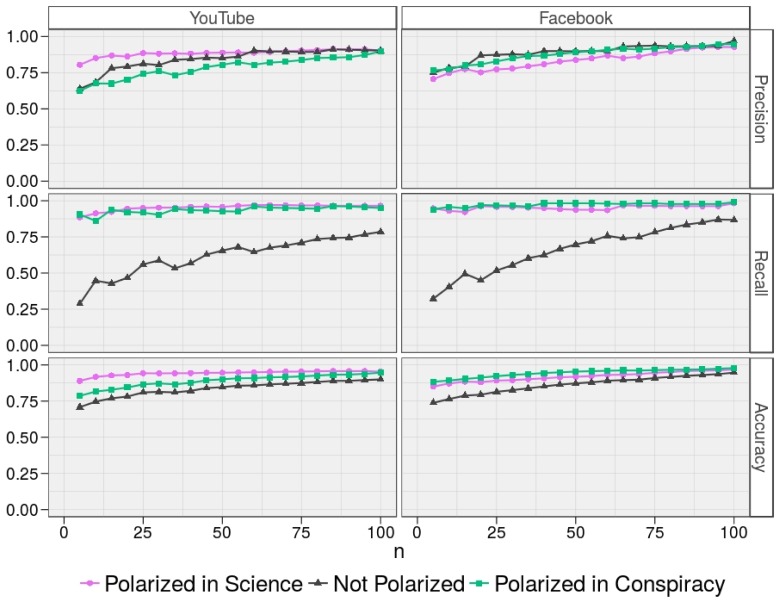
Performance measures the classification task. Precision, recall, and accuracy of the classification task for users Polarized in Conspiracy, Not Polarized, Polarized in Science on Facebook and YouTube as a function of *n*. On both online social networks, we find that the model’s performance measures monotonically increase as a function of *n*. Focusing on the accuracy, significant results (greater than 0.70) are obtained for low values of *n*.

Fig 6 shows precision, recall, and accuracy of the classification tasks on Facebook and YouTube as a function of *n*. On both online social networks, we find that the model’s performances monotonically increase as a function of *n* for each class. Focusing on accuracy, significant results (greater than 0.70) are obtained for low values of *n*. A suitable compromise between classification performances and required number of comments seems to be *n* = 50, which provides an accuracy greater than 0.80 for each class on both YouTube and Facebook. To assess how the results generalize to independent datasets and to limit problems like overfitting, we split YouTube and Facebook users datasets in training sets (*N* = 1000) and test sets (*N* = 200), and we perform Monte Carlo cross validations with 10^3^ iterations. Results of Monte Carlo validations are shown in Table 1 and confirm the goodness of the model.

**Table 1:** Monte Carlo Cross Validation. Mean and standard deviation (obtained averaging results of 10^3^ iterations) of precision, recall, and accuracy of the classification task for users Polarized in Conspiracy, Not Polarized, Polarized in Science.

	YouTube			Facebook		
	Precision	Recall	Accuracy	Precision	Recall	Accuracy
Polarized in Conspiracy	0.80 ± 0.04	0.93 ± 0.03	0.90 ± 0.02	0.89 ± 0.03	0.98 ± 0.02	0.95 ± 0.01
Not Polarized	0.85 ± 0.05	0.65 ± 0.06	0.85 ± 0.02	0.90 ± 0.04	0.70 ± 0.05	0.87 ± 0.02
Polarized in Science	0.89 ± 0.04	0.96 ± 0.02	0.95 ± 0.01	0.84 ± 0.04	0.94 ± 0.03	0.92 ± 0.02

We conclude that the early interaction of users with contents is an accurate predictor for the preferential attachment to a community and thus for the emergence of echo chambers. Moreover, in Table 2, we show that the evolution of the polarization on Facebook and YouTube is so alike that the same model (with *n* = 50), when trained with Facebook users (*N* = 1200) to classify YouTube users (*N* = 1200), leads to an accuracy in the classification task greater than 0.80 for each class. Similarly, using YouTube users as training set to classify Facebook users leads to similar performances.

**Table 2:** Performance measures of classification. Precision, recall, and accuracy of the classification task for users Polarized in Conspiracy, Not Polarized, Polarized in Science when YouTube users are used as training set to classify Facebook users (top table), and when Facebook users are used as training set to classify YouTube users (bottom table).

**Training YouTube—Test Facebook**			
	Precision	Recall	Accuracy
Polarized in Conspiracy	0.90	0.95	0.95
Not Polarized	0.90	0.41	0.79
Polarized in Science	0.68	1.00	0.84
**Training Facebook—Test YouTube**			
Polarized in Conspiracy	0.77	0.96	0.89
Not Polarized	0.72	0.69	0.81
Polarized in Science	0.97	0.77	0.91

## Conclusions

Algorithms for content promotion are supposed to be the main determinants of the polarization effect arising out of online social media. Still, not much is known about the role of cognitive factors in driving users to aggregate in echo chambers supporting their favorite narrative. Recent studies suggest confirmation bias as one of the driving forces of content selection, which eventually leads to the emergence of polarized communities [12–15].

Our findings show that conflicting narratives lead to the aggregation of users in homogeneous echo chambers, irrespective of the online social network and the algorithm of content promotion.

Indeed, in this work, we characterize the behavioral patterns of users dealing with the same contents, but different mechanisms of content promotion. In particular, we investigate whether different mechanisms regulating content promotion in Facebook and Youtube lead to the emergence of homogeneous echo chambers.

We study how users interact with two very distinct and conflicting narratives—i.e. conspiracy-like and scientific news—on Facebook and YouTube. Using extensive quantitative analysis, we find the emergence of polarized and homogeneous communities supporting competing narratives that behave similarly on both online social networks. Moreover, we analyze the evolution of polarization, i.e. how users become polarized towards a narrative. Still, we observe strong similarities between behavioral patterns of users supporting conflicting narratives on different online social networks.

Such a common behavior allows us to derive a statistical learning model to predict with a good precision whether a user will become polarized towards a certain narrative or she will continue to switch between contents supporting different narratives. Finally, we observe that the behavioral patterns are so similar in Facebook and YouTube that we are able to predict with a good precision the polarization of Facebook users by training the model with YouTube users, and vice versa.

## Methods

### Ethics Statement

The entire data collection process has been carried out exclusively through the Facebook Graph API [23] and the YouTube Data API [24], which are both publicly available, and for the analysis we used only public available data (users with privacy restrictions are not included in the dataset). The pages from which we download data are public Facebook and YouTube entities. User content contributing to such entities is also public unless the user’s privacy settings specify otherwise and in that case it is not available to us. We abided by the terms, conditions, and privacy policies of the websites (Facebook/Youtube)

### Data Collection

The Facebook dataset is composed of 413 US public pages divided to Conspiracy and Science news. The first category (Conspiracy) includes pages diffusing alternative information sources and myth narratives—pages which disseminate controversial information, usually lacking supporting evidence and most often contradictory of the official news. The second category (Science) includes scientific institutions and scientific press having the main mission of diffusing scientific knowledge. Such a space of investigation is defined with the same approach as in [19], with the support of different Facebook groups very active in monitoring the conspiracy narratives. Pages were accurately selected and verified according to their self description. For both the categories of pages we downloaded all the posts (and their respective users interactions) in a timespan of 5 years (Jan 2010 to Dec 2014). To our knowledge, the final dataset is the complete set of all scientific and conspiracy-like information sources active in the US Facebook scenario up to date.

We pick all posts on Facebook linking a video on Youtube and then through the API we downloaded the videos related metadata. To build the Youtube database of video we downloaded likes, comments and descriptions of each video cited/shared in Facebook posts using the Youtube Data API [25]. Each video link in Facebook contains an unique id that identify the resource in a unique way on both Facebook and Youtube. The comments thread in Youtube, with its time sequence, is the equivalent of the feed timeline in a Facebook page. The techniques used to analyse Facebook data can be then used in Youtube data with minimum modifications. The YouTube dataset is composed of about 17*K* videos linked by Facebook posts supporting Science or Conspiracy news. Videos linked by posts in Science pages are considered as videos disseminating scientific knowledge, whereas videos linked by posts in Conspiracy pages are considered as videos diffusing controversial information and supporting myth and conspiracy-like theories. Such a categorization is validated by all the authors and Facebook groups very active in monitoring conspiracy narratives. The exact breakdown of the data is shown in Tables 3, 4, 5 and 6. Summarizing, the dataset is composed of all public videos posted by the Facebook pages listed in the Page List section and their related instances on Youtube.

**Table 3:** Breakdown of the dataset.

	**Facebook**		
	Science	Conspiracy	Total
Posts	4,388	16,689	21,077
Likes	925*K*	1*M*	1.9*M*
Comments	86*K*	127*K*	213*K*
Shares	312*K*	493*K*	805*K*
	**YouTube**		
	Science	Conspiracy	Total
Videos	3,803	13,649	17,452
Likes	13.5*M*	31*M*	44.5*M*
Comments	5.6*M*	11.2*M*	16.8*M*
Views	2.1*M*	6.33*M*	8.41*M*

**Table 4:** Conspiracy Pages.

	Page Name	Facebook ID
1	Spirit Science and Metaphysics	171274739679432
2	Spirit Science	210238862349944
3	The Conspiracy Archives	262849270399655
4	iReleaseEndorphins	297719273575542
5	World of Lucid Dreaming	98584674825
6	The Science of Spirit	345684712212932
7	Esoteric Philosophy	141347145919527
8	9/11 Truth Movement	259930617384687
9	Great Health The Natural Way	177320665694370
10	New World Order News	111156025645268
11	Freedom Isn’t Free on FB	634692139880441
12	Skeptic Society	224391964369022
13	The Spiritualist	197053767098051
14	Anonymous World Wide	494931210527903
15	The Life Beyond Earth	152806824765696
16	Illuminati Exposed	298088266957281
17	Illuminating Souls	38466722555
18	Alternative Way	119695318182956
19	Paranormal Conspiracies	455572884515474
20	CANNABIS CURES CANCERS!	115759665126597
21	Natural Cures Not Medicine	1104995126306864
22	CTA Conspiracy Theorists’ Association	515416211855967
23	Illuminati Killers	478715722175123
24	Conspiracy 2012 & Beyond	116676015097888
25	GMO Dangers	182443691771352
26	The Truthers Awareness	576279865724651
27	Exposing the truth about America	385979414829070
28	Occupy Bilderberg	231170273608124
29	Speak the Revolution	422518854486140
30	I Don’t Trust The Government	380911408658563
31	Sky Watch Map	417198734990619
32	| truthaholics	201546203216539
33	UFO Phenomenon	419069998168962
34	Conspiracy Theories & The Illuminati	117611941738491
35	Lets Change The World	625843777452057
36	Makaveli The Prince Killuminati	827000284010733
37	It’s A New Day	116492031738006
38	New world outlawz—killuminati soldiers	422048874529740
39	The Government’s bullshit. Your argument is invalid.	173884216111509
40	America Awakened	620954014584248
41	The truth behold	466578896732948
42	Alien Ufo And News	334372653327841
43	Anti-Bilderberg Resistance Movement	161284443959494
44	The Truth Unleashed	431558836898020
45	Anti GMO Foods and Fluoride Water	366658260094302
46	STOP Controlling Nature	168168276654316
47	9/11 Blogger	109918092364301
48	9/11 Studies and Outreach Club at ASU	507983502576368
49	9/11 Truth News	120603014657906
50	Abolish the FDA	198124706875206
51	AboveTopSecret.com	141621602544762
52	Activist Post	128407570539436
53	Alliance for Natural Health USA	243777274534
54	All Natural & Organic. Say No To Toxic Chemicals.	323383287739269
55	Alternative Medicine	219403238093061
56	Alternative World News Network	154779684564904
57	AltHealthWORKS	318639724882355
58	American Academy of Environmental Medicine	61115567111
59	American Association of Naturopathic Physicians	14848224715
60	Ancient Alien Theory	147986808591048
61	Ancient Aliens	100140296694563
62	Ancient Astronaut Theory	73808938369
63	The Anti-Media	156720204453023
64	Anti Sodium Fluoride Movement	143932698972116
65	Architects & Engineers for 9/11 Truth	59185411268
66	Association of Accredited Naturopathic Medical Colleges (AANMC)	60708531146
67	Autism Media Channel	129733027101435
68	Babes Against Biotech	327002374043204
69	Bawell Alkaline Water Ionizer Health Benefits	447465781968559
70	CancerTruth	348939748204
71	Chemtrails Awareness	12282631069
72	Collective Evolution	131929868907
73	Conspiracy Theory With Jesse Ventura	122021024620821
74	The Daily Sheeple	114637491995485
75	Dr. Bronner’s Magic Soaps	33699882778
76	Dr. Joseph Mercola	114205065589
77	Dr. Ronald Hoffman	110231295707464
78	Earth. We are one.	149658285050501
79	Educate Inspire Change	467083626712253
80	Energise for Life: The Alkaline Diet Experts!	99263884780
81	Exposing The Truth	175868780941
82	The Farmacy	482134055140366
83	Fluoride Action Network	109230302473419
84	Food Babe	132535093447877
85	Global Research (Centre for Research on Globalization)	200870816591393
86	GMO Inside	478981558808326
87	GMO Just Say No	1390244744536466
88	GreenMedInfo.com	111877548489
89	Healthy Holistic Living	134953239880777
90	I Fucking Love Truth	445723122122920
91	InfoWars	80256732576
92	Institute for Responsible Technology	355853721234
93	I Want To Be 100% Organic	431825520263804
94	Knowledge of Today	307551552600363
95	La Healthy Living	251131238330504
96	March Against Monsanto	566004240084767
97	Millions Against Monsanto by OrganicConsumers.org	289934516904
98	The Mind Unleashed	432632306793920
99	Moms Across America	111116155721597
100	Moms for Clean Air/Stop Jet Aerosol Spraying	1550135768532988
101	Natural Society	191822234195749
102	Non-GMO Project	55972693514
103	Occupy Corporatism	227213404014035
104	The Open Mind	782036978473504
105	Organic Consumers Association	13341879933
106	Organic Health	637019016358534
107	The Organic Prepper	435427356522981
108	PreventDisease.com	199701427498
109	Raw For Beauty	280583218719915
110	REALfarmacy.com	457765807639814
111	ReThink911	581078305246370
112	Sacred Geometry and Ancient Knowledge	363116270489862
113	Stop OC Smart Meters	164620026961366
114	The Top Information Post	505941169465529
115	The Truth About Vaccines	133579170019140
116	Truth Teller	278837732170258
117	Veterans Today	170917822620
118	What Doctors Don’t Tell You	157620297591924
119	Wheat Belly	209766919069873
120	Why don’t you try this?	202719226544269
121	WND	119984188013847
122	WorldTruth.TV	114896831960040
123	Zeitgeist	32985985640
124	Ancient Origins	530869733620642
125	Astrology Answers	413145432131383
126	Astrology News Service	196416677051124
127	Autism Action Network	162315170489749
128	Awakening America	406363186091465
129	Awakening People	204136819599624
130	Cannabinoids Cure Diseases & The Endocannabinoid System Makes It Possible.	322971327723145
131	Celestial Healing Wellness Center	123165847709982
132	Chico Sky Watch	149772398420200
133	A Conscious awakening	539906446080416
134	Conspiracy Syndrome	138267619575029
135	Conspiracy Theory: Truth Hidden in Plain Sight, and Army of SATAN	124113537743088
136	Cosmic Intelligence-Agency	164324963624932
137	C4ST	371347602949295
138	Deepak Chopra	184133190664
139	Dr. Mehmet Oz	35541499994
140	Earth Patriot	373323356902
141	Electromagnetic Radiation Safety	465980443450930
142	EMF Safety Network	199793306742863
143	End Time Headlines	135010313189665
144	Young Living Essential Oils	29796911981
145	Exposing Bilderberg 2012	300498383360728
146	Exposing The Illuminati	196087297165394
147	Exposing Satanic World Government	529736240478567
148	FEMA Camps Exposed	285257418255898
149	Fight Against Illuminati And New World Order	195559810501401
150	FitLife.tv	148518475178805
151	GMO Free USA	402058139834655
152	Holistic Health	105497186147476
153	The Illuminati	543854275628660
154	Illuminati Mind Control	499866223357022
155	Intelwars	130166550361356
156	Natural Solutions Foundation	234136166735798
157	NWO Truth Radio	135090269995781
158	Occupy Bilderberg 2012	227692450670795
159	Operation: Awakening- The Global Revolution	287772794657070
160	The Paradigm Shift	221341527884801
161	PositiveMed	177648308949017
162	Press TV	145097112198751
163	The Resistance	394604877344757
164	Rima E. Laibow, M.D.—Save My Life Dr. Rima	107527312740569
165	RT America	137767151365
166	Ruble’s Wonderings—Forbidden Archeology & Science	265422293590870
167	Seekers Of Truth	736499966368634
168	Spiritual Ecology	261982733906722
169	Spiritualer.com	531950866874307
170	Take Back Your Power	269179579827247
171	There is a cure for Cancer, but it is not FDA approved. Phoenix Tears work!	395190597537
172	True Activist	129370207168068
173	Truth Exposed Radio	173823575962481
174	Truth Movement	161389033958012
175	Truth Network	271701606246002
176	Wake up call	276404442375280
177	We Should Ban GMOs	516524895097781
178	vactruth.com	287991907988
179	Veterans Today Truth Warriors	645478795537771
180	4 Foot Farm Blueprint	1377091479178258
181	Dawning Golden Crystal Age	127815003927694
182	Occupy Your Mind	393849780700637
183	We do not Forgive. We do not Forget. We are Anonymous. Expect Us.	134030470016833
184	Health Impact News	469121526459635
185	NaturalNews.com	35590531315
186	World for 9/11 Truth	38411749990
187	Beware of Disinformation	558882824140805
188	Citizens For Legitimate Government	93486533659
189	Cureyourowncancer.org	535679936458252
190	Juicing Vegetables	172567162798498
191	Quantum Prophecies	323520924404870
192	AIM Integrative Medicine	137141869763519
193	Autism Nutrition Research Center	1508552969368252
194	The Canary Party	220071664686886
195	Chemtrail Research	247681531931261
196	Chemtrail Watchers	77065926441
197	Children’s Medical Safety Research Institute	790296257666848
198	Contaminated Vaccines	686182981422650
199	Dane Wigington	680418385353616
200	David Icke	147823328841
201	David Icke Books Limited	191364871070270
202	David Icke—Headlines	1421025651509652
203	Disinformation Directory	258624097663749
204	The Drs. Wolfson	1428115297409777
205	Educate, Inspire & Change. The Truth Is Out There, Just Open Your Eyes	111415972358133
206	Focus for Health Foundation	456051981200997
207	Generation Rescue	162566388038
208	Geoengineering Watch	448281071877305
209	Global Skywatch	128141750715760
210	The Greater Good	145865008809119
211	The Health Freedom Express	450411098403289
212	Homegrown Health	190048467776279
213	Intellihub	439119036166643
214	The Liberty Beacon	222092971257181
215	International Medical Council on Vaccination	121591387888250
216	International Medical Council on Vaccination—Maine Chapter	149150225097217
217	Medical Jane	156904131109730
218	Mississippi Parents for Vaccine Rights	141170989357307
219	My parents didn’t put me in time-out, they whooped my ass!	275738084532
220	National Vaccine Information Center	143745137930
221	The Raw Feed Live	441287025913792
222	Rinf.com	154434341237962
223	SANEVAX	139881632707155
224	Things pro-vaxers say	770620782980490
225	Unvaccinated America	384030984975351
226	Vaccine Injury Law Project	295977950440133
227	Vermont Coalition for Vaccine Choice	380959335251497
228	9/11: The BIGGEST LIE	129496843915554
229	Agent Orange Activists	644062532320637
230	Age of Autism	183383325034032
231	AutismOne	199957646696501
232	Awakened Citizen	481936318539426
233	Best Chinese Medicines	153901834710826
234	Black Salve	224002417695782
235	Bought Movie	144198595771434
236	Children Of Vietnam Veterans Health Alliance	222449644516926
237	Collective-Evolution Shift	277160669144420
238	Doctors Are Dangerous	292077004229528
239	Dr. Tenpenny on Vaccines	171964245890
240	Dr Wakefield’s work must continue	84956903164
241	EndoRIOT	168746323267370
242	Enenews	126572280756448
243	Expanded Consciousness	372843136091545
244	Exposing the truths of the Illuminati II	157896884221277
245	Family Health Freedom Network	157276081149274
246	Fearless Parent	327609184049041
247	Food Integrity Now	336641393949
248	Four Winds 10	233310423466959
249	Fukushima Explosion What You Do Not Know	1448402432051510
250	The Golden Secrets	250112083847
251	Health Without Medicine & Food Without Chemicals	304937512905083
252	Higher Perspective	488353241197000
253	livingmaxwell	109584749954
254	JFK Truth	1426437510917392
255	New World Order Library | NWO Library	194994541179
256	No Fluoride	117837414684
257	Open Minds Magazine	139382669461984
258	Organic Seed Alliance	111220277149
259	Organic Seed Growers and Trade Association	124679267607065
260	RadChick Radiation Research & Mitigation	260610960640885
261	The REAL Institute—Max Bliss	328240720622120
262	Realities Watch	647751428644641
263	StormCloudsGathering	152920038142341
264	Tenpenny Integrative Medical Centers (TIMC)	144578885593545
265	Vaccine Epidemic	190754844273581
266	VaccineImpact	783513531728629
267	Weston A. Price Foundation	58956225915
268	What On Earth Is Happening	735263086566914
269	The World According to Monsanto	70550557294
270	Truth Theory	175719755481
271	Csglobe	403588786403016
272	Free Energy Truth	192446108025
273	Smart Meter Education Network	630418936987737
274	The Mountain Astrologer magazine	112278112664
275	Alberta Chemtrail Crusaders	1453419071541217
276	Alkaline Us	430099307105773
277	Americas Freedom Fighters	568982666502934
278	Anti-Masonic Party Founded 1828	610426282420191
279	Cannabidiol OIL	241449942632203
280	Cancer Compass An Alternate Route	464410856902927
281	Collective Evolution Lifestyle	1412660665693795
282	Conscious Life News	148270801883880
283	Disclosure Project	112617022158085
284	Dr. Russell Blaylock, MD	123113281055091
285	Dumbing Down People into Sheeple	123846131099156
286	Expand Your Consciousness	351484988331613
287	Fluoride: Poison on Tap	1391282847818928
288	Gaiam TV	182073298490036
289	Gary Null & Associates	141821219197583
290	Genesis II Church of Health & Healing (Official)	115744595234934
291	Genetic Crimes Unit	286464338091839
292	Global Healing Center	49262013645
293	Gluten Free Society	156656676820
294	GMO Free Oregon	352284908147199
295	GMO Journal	113999915313056
296	GMO OMG	525732617477488
297	GreenMedTV	1441106586124552
298	Healing The Symptoms Known As Autism	475607685847989
299	Health Conspiracy Radio	225749987558859
300	Health and Happiness	463582507091863
301	Jesse Ventura	138233432870955
302	Jim Humble	252310611483446
303	Kid Against Chemo	742946279111241
304	Kids Right To Know Club	622586431101931
305	The Master Mineral Solution of the 3rd Millennium	527697750598681
306	Millions Against Monsanto Maui	278949835538988
307	Millions Against Monsanto World Food Day 2011	116087401827626
308	Newsmax Health	139852149523097
309	Non GMO journal	303024523153829
310	Nurses Against ALL Vaccines	751472191586573
311	Oath Keepers	182483688451972
312	Oath Keepers of America	1476304325928788
313	The Organic & Non-GMO Report	98397470347
314	Oregon Coast Holographic Skies Informants	185456364957528
315	Paranormal Research Project	1408287352721685
316	Politically incorrect America	340862132747401
317	(Pure Energy Systems) PES Network, Inc.	183247495049420
318	Save Hawaii from Monsanto	486359274757546
319	Sayer Ji	205672406261058
320	SecretSpaceProgram	126070004103888
321	SPM Southern Patriots MIlitia	284567008366903
322	Thrive	204987926185574
323	Truth Connections	717024228355607
324	Truth Frequency	396012345346
325	Truthstream Media.com	193175867500745
326	VT Right To Know GMOs	259010264170581
327	We Are Change	86518833689
328	Wisdom Tribe 7 Walking in Wisdom.	625899837467523
329	World Association for Vaccine Education	1485654141655627
330	X Tribune	1516605761946273

**Table 5:** Science Pages.

	Page Name	Facebook ID
1	AAAS—The American Association for the Advancement of Science	19192438096
2	AAAS Dialogue on Science, Ethics and Religion	183292605082365
3	Armed with Science	228662449288
4	AsapSCIENCE	162558843875154
5	Bridge to Science	185160951530768
6	EurekAlert!	178218971326
7	Food Science	165396023578703
8	Food Science and Nutrition	117931493622
9	I fucking love science	367116489976035
10	LiveScience	30478646760
11	Medical Laboratory Science	122670427760880
12	National Geographic Magazine	72996268335
13	National Science Foundation (NSF)	30037047899
14	Nature	6115848166
15	Nature Education	109424643283
16	Nature Reviews	328116510545096
17	News from Science	100864590107
18	Popular Science	60342206410
19	RealClearScience	122453341144402
20	Science	96191425588
21	Science and Mathematics	149102251852371
22	Science Channel	14391502916
23	Science Friday	10862798402
24	Science News Magazine	35695491869
25	Science-Based Medicine	354768227983392
26	Science-fact	167184886633926
27	Science, Critical Thinking and Skepticism	274760745963769
28	Science: The Magic of Reality	253023781481792
29	ScienceDaily	60510727180
30	ScienceDump	111815475513565
31	ScienceInsider	160971773939586
32	Scientific American magazine	22297920245
33	Scientific Reports	143076299093134
34	Sense About Science	182689751780179
35	Skeptical Science	317015763334
36	The Beauty of Science & Reality.	215021375271374
37	The Flame Challenge	299969013403575
38	The New York Times—Science	105307012882667
39	Wired Science	6607338526
40	All Science, All the Time	247817072005099
41	Life’s Little Mysteries	373856446287
42	Reason Magazine	17548474116
43	Nature News and Comment	139267936143724
44	Astronomy Magazine	108218329601
45	CERN	169005736520113
46	Citizen Science	200725956684695
47	Cosmos	143870639031920
48	Discover Magazine	9045517075
49	Discovery News	107124643386
50	Genetics and Genomics	459858430718215
51	Genetic Research Group	193134710731208
52	Medical Daily	189874081082249
53	MIT Technology Review	17043549797
54	NASA—National Aeronautics and Space Administration	54971236771
55	New Scientist	235877164588
56	Science Babe	492861780850602
57	ScienceBlogs	256321580087
58	Science, History, Exploration	174143646109353
59	Science News for Students	136673493023607
60	The Skeptics Society & Skeptic Magazine	23479859352
61	Compound Interest	1426695400897512
62	Kevin M. Folta	712124122199236
63	Southern Fried Science	411969035092
64	ThatsNonsense.com	107149055980624
65	Science & Reason	159797170698491
66	ScienceAlert	7557552517
67	Discovery	6002238585
68	Critical Thinker Academy	175658485789832
69	Critical Thinking and Logic Courses in US Core Public School Curriculum	171842589538247
70	Cultural Cognition Project	287319338042474
71	Foundation for Critical Thinking	56761578230
72	Immunization Action Coalition	456742707709399
73	James Randi Educational Foundation	340406508527
74	NCSE: The National Center for Science Education	185362080579
75	Neil deGrasse Tyson	7720276612
76	Science, Mother Fucker. Science	228620660672248
77	The Immunization Partnership	218891728752
78	Farm Babe	1491945694421203
79	Phys.org	47849178041
80	Technology Org	218038858333420
81	Biology Fortified, Inc.	179017932138240
82	The Annenberg Public Policy Center of the University of Pennsylvania	123413357705549
83	Best Food Facts	200562936624790

**Table 6:** Debunking Pages.

	Page Name	Facebook ID
1	Refutations to Anti-Vaccine Memes	414643305272351
2	Boycott Organic	1415898565330025
3	Contrails and Chemtrails:The truth behind the myth	391450627601206
4	Contrail Science	339553572770902
5	Contrail Science and Facts—Stop the Fear Campaign	344100572354341
6	Debunking Denialism	321539551292979
7	The Farmer’s Daughter	350270581699871
8	GMO Answers	477352609019085
9	The Hawaii Farmer’s Daughter	660617173949316
10	People for factual GMO truths (pro-GMO)	255945427857439
11	The Questionist	415335941857289
12	Scientific skepticism	570668942967053
13	The Skeptic’s Dictionary	195265446870
14	Stop the Anti-Science Movement	1402181230021857
15	The Thinking Person’s Guide to Autism	119870308054305
16	Antiviral	326412844183079
17	Center for Inquiry	5945034772
18	The Committee for Skeptical Inquiry	50659619036
19	Doubtful News	283777734966177
20	Hoax-Slayer	69502133435
21	I fucking hate pseudoscience	163735987107605
22	The Genetic Literacy Project	126936247426054
23	Making Sense of Fluoride	549091551795860
24	Metabunk	178975622126946
25	Point of Inquiry	32152655601
26	Quackwatch	220319368131898
27	Rationalwiki	226614404019306
28	Science-Based Pharmacy	141250142707983
29	Skeptical Inquirer	55675557620
30	Skeptic North	141205274247
31	The Skeptics’ Guide to the Universe	16599501604
32	Society for Science-Based Medicine	552269441534959
33	Things anti-vaxers say	656716804343725
34	This Week in Pseudoscience	485501288225656
35	Violent metaphors	537355189645145
36	wafflesatnoon.com	155026824528163
37	We Love GMOs and Vaccines	1380693538867364
38	California Immunization Coalition	273110136291
39	Exposing PseudoAstronomy	218172464933868
40	CSICOP	157877444419
41	The Panic Virus	102263206510736
42	The Quackometer	331993286821644
43	Phil Plait	251070648641
44	Science For The Open Minded	274363899399265
45	Skeptic’s Toolbox	142131352492158
46	Vaccine Nation	1453445781556645
47	Vaximom	340286212731675
48	Voices for Vaccines	279714615481820
49	Big Organic	652647568145937
50	Chemtrails are NOT real, idiots are.	235745389878867
51	Sluts for Monsanto	326598190839084
52	Stop Homeopathy Plus	182042075247396
53	They Blinded Me with Pseudoscience	791793554212187
54	Pro-Vaccine Shills for Big Pharma, the Illumanati, Reptilians, and the NWO	709431502441281
55	Pilots explain Contrails—and the Chemtrail Hoax	367930929968504
56	The Skeptical Beard	325381847652490
57	The Alliance For Food and Farming	401665083177817
58	Skeptical Raptor	522616064482036
59	Anti-Anti-Vaccine Campaign	334891353257708
60	Informed Citizens Against Vaccination Misinformation	144023769075631
61	Museum of Scientifically Proven Supernatural and Paranormal Phenomena	221030544679341
62	Emergent	375919272559739
63	Green State TV	128813933807183
64	Kavin Senapathy	1488134174787224
65	vactruth.com Exposed	1526700274269631
66	snopes.com	241061082705085

### Preliminaries and Definitions

#### Polarization of Users

Polarization of users, *ρ*_*u*_ ∈ [0, 1], is defined as the fraction of comments that a user *u* left on posts (videos) supporting conspiracy-like narratives on Facebook (YouTube). In mathematical terms, given *s*_*u*_, the number of comments left on Science posts by user *u*, and *c*_*u*_, the number of comments left on Conspiracy posts by user *u*, the polarization of *u* is defined as

$$\rho_{u}=\frac{c_{u}}{s_{u}+c_{u}}.$$

We then consider users with *ρ*_*u*_ > 0.95 as users polarized towards Conspiracy, and users with *ρ*_*u*_ < 0.05 as users polarized towards Science.

#### Bimodality Coefficient

The Bimodality Coefficient (BC) [26] is defined as

$$BC=\frac{\mu_{3}^{2}+1}{\mu_{4}+3\frac{{\left(n-1\right)}^{2}}{\left(n-2)(n-3\right)}},$$

with *μ*_3_ referring to the skewness of the distribution and *μ*_4_ referring to its excess kurtosis, with both moments being corrected for sample bias using the sample size *n*.

The BC of a given empirical distribution is then compared to a benchmark value of *BC*_*crit*_ = 5/9 ≈ 0.555 that would be expected for a uniform distribution; higher values point towards bimodality, whereas lower values point toward unimodality.

#### Multinomial Logistic Model

Multinomial logistic regression is a classification method that generalizes logistic regression to multi-class problems, i.e. with more than two possible discrete outcomes [27]. Such a model is used to predict the probabilities of the different possible outcomes of a categorically distributed dependent variable, given a set of independent variables. In the multinomial logistic model we assume that the log-odds of each response follow a linear model

$$\eta_{ij}=\log\left(\frac{\pi_{ij}}{\pi_{iJ}}\right)=\alpha_{j}+x_{i}^{T}\beta_{j},$$

where *α*_*j*_ is a constant and *β*_*j*_ is a vector of regression coefficients, for *j* = 1, 2, …, *J* − 1. Such a model is analogous to a logistic regression model, except that the probability distribution of the response is multinomial instead of binomial, and we have *J* − 1 equations instead of one. The *J* − 1 multinomial logistic equations contrast each of categories *j* = 1, 2, …, *J* − 1 with the baseline category *J*. If *J* = 2 the multinomial logistic model reduces to the simple logistic regression model.

The multinomial logistic model may also be written in terms of the original probabilities *π*_*ij*_ rather than the log-odds. Indeed, assuming that *η*_*iJ*_ = 0, we can write

$$\pi_{ij}=\frac{\exp(\eta_{ij})}{\sum_{k=1}^{J}\exp\left(\eta_{ik}\right)}.$$

#### Classification Performance Measures

To assess the goodness of our model we use three different measures of classification performance: precision, recall, and accuracy. For each class *i*, we compute the number of true positive cases *TP*_*i*_, true negative cases *TN*_*i*_, false positive cases *FP*_*i*_, and false negative cases *FN*_*i*_. Then, for each class *i* the precision of the classification is defined as

$$precision_{i}=\frac{TP_{i}}{TP_{i}+FP_{i}},$$

the recall is defined as

$$recall_{i}=\frac{TP_{i}}{TP_{i}+FN_{i}},$$

and the accuracy is defined as

$$accuracy_{i}=\frac{TP_{i}+TN_{i}}{TP_{i}+TN_{i}+FP_{i}+FN_{i}}.$$

#### Power law distributions

Scaling exponents of power law distributions are estimated via maximum likelihood (ML) as shown in [28]. To provide a full probabilistic assessment about whether two distributions are similar, we estimate the posterior distribution of the difference between the scaling exponents through an Empirical Bayes method.

Suppose we have two samples of observations, *A* and *B*, following power law distributions. For the sample *A*, we use the ML estimate of the scaling parameter, ${\hat{\theta }}_{A}^{ML}$, as location hyper-parameter of a Normal distribution with scale hyper-parameter ${\hat{\sigma }}_{A}^{ML}$. Such a Normal distribution represents the prior distribution, $p\left(\theta_{A}\right)∼N\left({\hat{\theta }}_{A}^{ML},{\hat{\sigma }}_{A}^{ML}\right)$, of the scaling exponent *θ*_*A*_. Then, according to the Bayesian paradigm, the prior distribution, *p*(*θ*_*A*_), is updated into a posterior distribution, *p*(*θ*_*A*_|*x*_*A*_):

$$p\left(\theta_{A}|x_{A}\right)=\frac{p\left(x_{A}|\theta_{A}\right)p\left(\theta_{A}\right)}{p(x_{A})},$$

where *p*(*x*_*A*_|*θ*_*A*_) is the likelihood. The posterior distribution is obtained via Metropolis-Hastings algorithm, i.e. a Markov Chain Monte Carlo (MCMC) method used to obtain a sequence of random samples from a probability distribution for which direct sampling is difficult [29–31]. To obtain reliable posterior distributions, we run 50,000 iterations (5,000 burned), which proved to ensure the convergence of the MCMC algorithm.

The posterior distribution of *θ*_*B*_ can be computed following the same steps. Once both posterior distributions, *p*(*θ*_*A*_|*x*_*A*_) and *p*(*θ*_*B*_|*x*_*B*_), are derived, we compute the distribution of the difference between the scaling exponents by subtracting the posteriors, i.e.

$$p\left(\theta_{A}-\theta_{B}|x_{A},x_{B}\right)=p\left(\theta_{A}|x_{A}\right)-p\left(\theta_{B}|x_{B}\right).$$

Then, by observing the 90% High Density Interval (HDI90) of *p*(*θ*_*A*_ − *θ*_*B*_), we can draw a full probabilistic assessment of the similarity between the two distributions.
